# The Relationship Between Social Problem-Solving and Passive-Aggressive Behavior Among Adolescents

**DOI:** 10.3390/ejihpe15070140

**Published:** 2025-07-18

**Authors:** Zita Gál, Márió Tibor Nagy, István Károly Takács, László Kasik

**Affiliations:** 1 Institute of Psychology, University of Szeged, 6722 Szeged, Hungary; galzita@edu.u-szeged.hu; 2 School Failure Prevention Research Group, Hungarian Academy of Sciences–University of Szeged, 6722 Szeged, Hungary; 3 Institute of Education, University of Szeged, 6722 Szeged, Hungary; nagy.mario.tibor@edu.u-szeged.hu; 4 Doctoral School of Education, University of Szeged, 6722 Szeged, Hungary; f.takacs.istvan@edu.u-szeged.hu

**Keywords:** passive-aggressive behavior, social problem-solving, Hungarian adolescents, latent profile analysis

## Abstract

The aim of the study was to investigate the relationship between passive-aggressive behavior and social problem-solving among Hungarian adolescents (16- and 18-year-olds, N = 496). The Passive Aggression Scale (PAS) was used to explore the characteristics of criticism, ignoring, and sabotage, and the Social Problem-Solving Inventory–Revised (SPSI–R) was employed to measure negative and positive problem orientations and rational, impulsive, and avoidant problem-solving styles. Both questionnaires performed reliably for both ages. The results show that 18-year-olds are more likely to have a negative problem orientation. Both age groups show a similarly strong positive relationship between criticism–impulsivity and ignoring–rationality. Based on a latent profile analysis, two problem-solving profiles (mixed and positive rational) were distinguished for 16-year-olds and three (mixed, positive rational, and negative avoidant) for 18-year-olds. Only the profiles obtained for the older age groups differ in passive-aggressive characteristics: criticism is most common for impulsive avoiders, ignoring is least typical of positive rationalists, and sabotage is most characteristic of mixed profiles. Developmental and socio-psychological features are usually well understood in these patterns, but a measure of various passive-aggressive behaviors is needed that is specific to adolescents.

## 1. Introduction

Social competence is the psychological system of social (interpersonal) behavior, the functioning of its components (social and emotional skills, abilities, motives) is a fundamental determinant of social functioning and well-being at any age and has an impact not only on psychological well-being but also on academic success (Zsolnai, 2013). Social problem-solving and communication are components of social competence. Several studies have shown that their functioning during adolescence largely determines adulthood adaptability and well-being (e.g., Balogh-Pécsi et al., 2024; Romppanen et al., 2021; Zsolnai, 2013).

There are many empirically validated models of social (interpersonal) problem-solving, one of the most used being that of T. J. D’Zurilla et al. (2002). According to this model, social problem-solving is a motivational, cognitive, emotional, and behavioral process aimed at dealing with a situation or relationship that is perceived as a problem—one that triggers negative feelings and thoughts. In the orientation phase of the process, we assess our efficacy and emotional state and formulate our attitude towards the situation and the other person, which can be positive or negative, mainly in terms of self-efficacy and frustration tolerance (Chang et al., 2004). The orientation subprocess is the key determinant of the problem-solving subprocess, which can result in a variety of behavioral outcomes, depending on whether the individual focuses on the problem at hand (rational style), reduces negative emotions and mostly aims to achieve his/her own goals (impulsive style), or does not want to be part of the solution: he/she starts to solve the problem but then withdraws from the situation or does not even start to solve the problem at all (avoidant style) (J. T. D’Zurilla et al., 2004).

In general, positively oriented individuals show a rational problem-solving style, while negatively oriented individuals show an impulsive or avoidant style, and these may vary within a problem situation (Chang et al., 2004; Nezu et al., 2004). However, other combinations are possible. For example, the relationship between a positive problem orientation and avoidant style shows that an individual makes a very firm, well-reasoned decision about why he/she should not solve an interpersonal problem, or he/she is basically negatively oriented but bases his/her decision on facts, analyzing what would be the most ideal solution for himself/herself (Kasik, 2015). Data from both Hungarian and international studies (e.g., Chang et al., 2004; Kasik, 2012, 2015) show that during adolescence, in addition to significant individual differences due to personality and environmental determinants, negative problem orientation gradually increases, and impulsivity is high at this age. Avoidant styles are most common among 14–16-year-olds, with lower levels before and after this age. Rationality shows a significant relation with some cognitive abilities (e.g., inductive reasoning), thus making the developmental trajectory highly variable and painting a highly variable picture of positive problem orientation, where the influence of environmental factors (e.g., parenting style) is strong.

There is little research on the communicative aspect of social problem-solving: what verbal and non-verbal means we use to express our intentions, decisions, and feelings in a problem situation and what communicative behavior we show. Although research to date suggests that social problem-solving styles (linked to the orientation subprocess) differ in their verbal and non-verbal means of communicative expression (Balogh-Pécsi et al., 2024; Erozkan, 2013), studies that focus on passive-aggressive communication are few and far between (Yavuz & Güzel, 2020). These studies interpret the relationship between the two areas in such a way that communication forms are the means of expressing problem solving. This is what we have carried out in this research.

Of the basic communicative behaviors (assertive, aggressive, passive-aggressive, passive, and submissive), assertiveness has been the subject of much research. Various school and non-school programs that develop problem and conflict management aim to make the characteristics of this form of communication more enduring (Erozkan, 2013; Zsolnai, 2013). Positively oriented, problem-focused individuals are generally assertive (Pipas & Jaradat, 2010). They can express their thoughts and needs calmly in difficult circumstances, respect the opinions of others, are curious about the other party’s intentions, and attempt to solve a problem taking those opinions and intentions into account (Gaumer Erickson et al., 2018). Balogh-Pécsi et al. (2024) investigated the relationship between the factors (positive and negative problem orientation and rational, impulsive, and avoidant problem-solving styles) measured by the SPSI–R (T. J. D’Zurilla et al., 2002) and the two dimensions of assertive communication (listening to others and expressing one’s own needs, feelings, and thoughts) measured by the AQ (Gaumer Erickson et al., 2016) in 11–13-year-olds. Their research showed that adolescents with a positive problem orientation and a rational style scored higher in both dimensions of communication than those with a negative problem orientation and impulsive and avoidant styles. Listening to others was strongly associated with positive problem orientation and expressing one’s own needs, feelings, and thoughts with rationality, while both associations were stronger for girls.

The purpose of aggression is to cause pain, harm or injury physically, psychologically or verbally (Fauzi et al., 2023; Pereira et al., 2022). According to Allen and Anderson (2017), aggressive behavior is defined as any behavior that actively or passively causes harm to oneself or others. Two basic forms of aggressive behavior are generally distinguished: overt (e.g., fighting, shouting) and covert (e.g., gossiping, bullying), which are the most common types of aggression among adolescents. In addition, physical aggression (e.g., assault or threat of assault), verbal aggression (e.g., verbal abuse, insults), relational aggression (e.g., harming someone’s peer relationships or status) and cyberaggression (Pereira et al., 2022). These forms of aggression can already be identified in adolescents and are determined by a combination of biological (e.g., hormonal changes, genetics), environmental (e.g., family problems, parental patterns, peer influence, peer patterns), and psychological factors (e.g., personality traits, mental disorders). A study conducted with adolescents (14–18 years old) did not find any relationship between age and any type of aggression. Psychological maturity was associated with both direct and indirect aggression, and self-reliance was the main predictor of indirect aggression. General psychological maturity was more related to indirect aggression in males, so the increased psychological maturity was associated with a greater decrease of indirect aggression. So, the results suggest that indirect aggression is more common among less mature adolescents (Morales-Vives et al., 2014).

Negatively oriented and impulsive individuals are less able to listen to others, as their focus is usually on reducing their own negative feelings and their negative thoughts are less likely to allow for a change of perspective (Siu & Shek, 2005). Impulsive problem-solvers may also be characterized by reactive (affective) and instrumental aggression. The former occurs as a rapid response to a threat and is unplanned, while the latter can be interpreted as a planned means to achieve a goal. However, a distinction must also be made between aggression of a kind whose aim is to protect others. Even among preschool children, behaviors in frustrating situations that are emotion-driven and aimed at changing the situation quickly without considering the other person’s intentions are well identified (Neel et al., 1990). At this age, self-centeredness is often a strong explanatory factor, but greater awareness is expected of the individual in problem situations in childhood and adolescence. Several studies have shown that it is precisely the level of awareness and control that is low in those who act aggressively when solving a peer problem. They often have confidence that aggression is an appropriate means of solving the problem and may also believe that their aggressive behavior is acceptable (Matthys & Lochman, 2005). Negatively oriented problem-solvers often report low self-esteem, which is positively correlated with aggressive behavior among adolescents (Muarifah et al., 2022).

Because boys, unlike girls, are socialized not to show their vulnerability in most societies, boys often avoid situations where they need to show or talk about their negative feelings (e.g., sadness and fear). One consequence of this is that their negative emotions often manifest themselves in the form of aggression (e.g., Craig et al., 2007). Girls are less likely to be (overtly) aggressive and are expected to behave in a more empathetic, cooperative, and helpful way, and this expectation is a fundamental determinant of parental goals (Ladd, 2005). However, girls also exhibit aggressive behaviors, although they are less overt and more covert, with some of these behaviors being highly deliberate partly because of the expectation noted above. Research by Lansford et al. (2012) suggests that boys aged 7–10 are more likely than girls their age to deal with their problems with physical aggression but that there are no gender differences in relational aggression. Moreover, adolescent boys have higher rates of overt aggression (e.g., name-calling, teasing, and fighting) than girls (Rozzaqyah et al., 2020).

The negative emotions that underlie aggression (e.g., tension and anger) can be expressed passively (e.g., silent resistance, sarcasm, gossip). Passive-aggressive behavior and indirect aggression are not the same, but similar phenomena. Indirect aggression is a behavior that does not manifest itself in the form of direct or physical aggression, but rather through social manipulation, for example, in some roundabout way (e.g., Kaukiainen et al., 1999). Björkqvist et al. (1992) examined 8-, 11-, and 15-year-olds across several forms of aggression. They found that indirect aggression (such as gossiping, breaking contact with the person) appears more frequently among girls. As for age differences, it is not yet fully developed at the age of 8. It is clearly typical among girls at the ages of 11 and 15. Assuming a relationship between indirect aggression and peer-rated social intelligence, they found that physical and direct aggression were not related to social intelligence among adolescents (10, 12, and 14 years of age). However, they found a relationship between indirect aggression and social intelligence, meaning that the more often an individual uses indirect aggression, the higher their social intelligence. The effective use of indirect aggression requires that the individual understand social relationships and skills (Kaukiainen et al., 1999).

Passive aggression allows an individual to express anger and related emotions without directly expressing these feelings to another. People who express passive aggression often deny that their behavior is hurtful (they have low insight). Passive-aggressive behavior is a difficult construct to examine in personality functioning (e.g., Millon et al., 2004; Pretzer & Beck, 1996). Severe forms of it were previously considered a personality disorder (Tringer, 2019). However, it has been found to be difficult to isolate at the diagnostic level and is not verifiable; it is thus no longer included in the DSM-5, which includes mental illness (Hopwood, 2018). The main reason for the change was that passive-aggressive functioning is a specific behavior pattern yet too narrow a category to be defined as a personality disorder. According to the previous DSM-IV (American Psychiatric Association, 1994), the core of passive-aggressive functioning was that the individual regularly procrastinates, shows resistance to authority figures, complains and feels sorry for himself/herself frequently, envies the success of others, and has difficulty finding a balance between aggressive and cooperative behavior. The definition of passive-aggressive behavior is far from simple. It describes a mode of functioning that is based on low self-esteem and manifests itself at the behavioral level in interpersonal conflicts, in both verbal and non-verbal forms, and can take many forms (Corcaci, 2022). Passive-aggressive functioning is essentially negative; that is, the person disagrees with something or does not want to do it but cannot or does not want to express his or her opposition clearly. The level of this resistance in everyday situations, including school situations, can range from unclear communication to procrastination to self-destructive behavior. It is expressed, for example, by some form of avoidance (ignoring, shutting out, delaying, and distancing), sabotage (appearing to agree and being helpful but then acting to the detriment of the other), criticism (lack of intention to improve and making prejudiced, disapproving remarks), hostility disguised as humor, and gossip (Schanz et al., 2021). Only some of these characteristics have been studied in adolescents, based on the assumption that they are already present at this age, but the process of the development of passive-aggressive behaviors in children and adolescents in a non-clinical sample is not well understood. The two-factor (self- and other-directed) questionnaire developed by Schanz et al. (2021) also measures characteristics of passive aggression in clinical and adult samples.

Passive-aggressive behavior is often characteristic of the negatively oriented and avoidant problem-solver. According to Schauenburg et al. (2007), this suggests that passive-aggressive behavior is an immature self-defense mechanism that suppresses emotional conflict and results in ineffective social problem-solving. According to Hopwood (2018), passive-aggressive behavior can be interpreted as a trait, is relatively stable, and can be stimulated by internal or external stressors—while a social problem situation can be interpreted as a stressor. For adolescents, it is sometimes very difficult to articulate negative feelings and identify the causes of a negative state, and, therefore, it is often difficult to find the most appropriate coping strategies. This shows poor rational problem-solving style and results in frequent avoidance (Özgür et al., 2011). Students who believe that passive-aggressive behavior is behavior that does not hurt others either physically or verbally, those students will tend to engage in passive-aggressive behavior because they believe that passive-aggressive can solve problems by avoiding the negative situation, thus suppressing negative feelings (Fauziah et al., 2024).

However, avoidant problem-solvers are not only passive-aggressive but also engage in passive and submissive behavior. The passive communicative person distances himself/herself from the problem situation and the other party, avoids him/her, and does not want to face the problem, while the submissive person subordinates his/her own needs and desires to the needs of others, often fearing humiliation (Balogh-Pécsi et al., 2024). Both Hungarian and international research suggests that the prevalence of avoidance increases in mid-adolescence (J. T. D’Zurilla et al., 2004; Kasik, 2015), but it can also have a myriad of behavioral manifestations. Research by Kasik et al. (2016) with 15- and 18-year-olds suggests that procrastination, gathering strength, and rumination are associated with positive problem orientation, as is asking for help; however, asking for help and problem-solving under external pressure (at the request of others and not of one’s own volition) are negatively associated with avoidance.

The aim of the research was to explore age-specific characteristics of social problem-solving and passive aggressive communication and age differences (sex differences within age), among 16- and 18-year-olds, and to use latent profile analysis to examine how groups that differ in social problem-solving differ along the characteristics of passive-aggressive behavior. This type of research has not yet been carried out in Hungary. Previous studies in Hungary have analyzed the relationship between social problem-solving and assertive communication, and based on this analysis, a developmental school program has been developed (Balogh-Pécsi et al., 2024). These studies and developments have not addressed passive-aggressive behavior. Based on the theoretical models and empirical research presented, we hypothesized the following: (1) avoidance is more typical of 16-year-olds, negative orientation is more typical of 18-year-olds, and there are no significant differences in positive problem orientation, impulsivity, and rationality (girls more typically have a negative problem orientation, and boys more commonly display avoidance at both ages) (Chang et al., 2004; Kasik, 2015); (2) since the expressions of passive-aggressive behavior being examined (criticism, ignoring, and sabotage) are most closely related to avoidance, they are more common for 16-year-olds (Lim & Suh, 2022; Özgür et al., 2011); and (3) a latent profile analysis allows us to distinguish different problem-solving profiles (attitude-style relationship), with different kinds of passive-aggressive behavior dominating (Fejes et al., 2023).

## 2. Methods

### 2.1. Participants

The study involved 16-year-olds (n = 260; M = 15.49, SD = 0.34) and 18-year-olds (n = 236; M = 17.78, SD = 0.68), with a total of 496 Hungarian adolescents (35.4% of the 16-year-olds and 42% of the 18-year-olds were girls). The reason for selecting 16-year-olds is that the avoidance rate is very high in this age period. We selected 18-year-olds because we have no data on the passive-aggressive behavioral characteristics of this age group in Hungary, only data on younger age groups are available (Balogh-Pécsi et al., 2024). The students had not participated in any emotional-social school development program in the year prior to the study. The two subsamples differed in terms of both parents’ educational level (1 = primary school, 2 = high school without high school diploma, 3 = high school with high school diploma, 4 = bachelor’s degree, 5 = master’s degree, 6 = PhD). The proportion of parents who had completed tertiary education is higher among mothers for the 16-year-olds and among fathers for the 18-year-olds (mothers: Pearson’s χ^2^ = 19.110, df = 5, *p* = 0.002; fathers: Pearson’s χ^2^ = 40.974, df = 5, *p* < 0.001).

### 2.2. Instruments

Two questionnaires were used in the study: (1) the Social Problem-Solving Inventory–Revised (SPSI–R, T. J. D’Zurilla et al., 2002) and (2) the Passive Aggression Scale (PAS, Lim & Suh, 2022).

*SPSI–R*. The questionnaire consists of 25 items grouped into five factors: (1) *positive problem orientation* (e.g., Solving a problem is a challenge for me), (2) *negative problem orientation* (e.g., When I have to make a decision, I feel nervous and uncertain), (3) *rationality* (e.g., When I have to solve a problem, the first thing I do is to learn as much as I can about it), (4) *impulsivity* (e.g., When I have to make a decision, I don’t think through the options carefully), and (5) *avoidance* (e.g., I do everything I can to avoid dealing with my problems). The statements are rated on a five-point scale (1 = Not at all true for me – 5 = Absolutely true for me). The reliability of the SPSI–R (Cronbach’s α) is appropriate at both ages (16-year-olds: 0.787; 18-year-olds: 0.780). The reliability of the SPSI–R (Cronbach’s α) is appropriate at both ages (16-year-olds: full questionnaire: 0.787, positive problem orientation: 0.731, negative problem orientation: 0.821, rationality: 0.754, impulsivity: 0.802, avoidance: 0.798; 18-year-olds: full questionnaire: 0.780; positive problem orientation: 0.755, negative problem orientation: 0.780, rationality: 0.794, impulsivity: 0.833, avoidance: 0.876).

*PAS*. The questionnaire consists of 21 items and measures three forms of passive-aggressive behavior: (1) *criticism* (e.g., When I talk about someone I dislike or find unpleasant, I pretend to praise their strengths but also drop hints about their weaknesses), (2) *ignoring* (e.g., I purposefully avoid eye contact with someone I don’t like or find unpleasant), and (3) *sabotage* (e.g., I come up with excuses and say things like ‘I forgot’ to someone I dislike or find unpleasant). Statements are rated on a five-point scale (1 = Almost never true for me–5 = Almost always true for me). The reliability of the PAS (Cronbach’s α) is good at both ages (16-year-olds: 0.882; 18-year-olds: 0.885). The reliability of the PAS (Cronbach’s α) is good at both ages (16-year-olds: full questionnaire: 0.882, criticism: 0.776, ignoring: 0.796, sabotage: 0.821; 18-year-olds: full questionnaire: 0.885, criticism: 0.797, ignoring: 0.821, sabotage: 0.856). This measure is used among adults (Lim & Suh, 2022), but there is no known instrument that measures passive aggressive behavior in adolescents in a non-clinical sample.

### 2.3. Data Collection and Analyses

The research was carried out in 2024. Students were given 1 teaching hour (45 min) to complete the two questionnaires and to provide the background variables (sex and parents’ educational level). Students were asked to think about their classmates and schoolmates while completing both questionnaires. The data collection was anonymous and it was supervised by psychology students who had been trained in data collection.

The data were analyzed with Jamovi 2.3.28 and SPSS 26. Cronbach’s α was chosen as the reliability indicator for the questionnaires. The data on the background variables were analyzed with the χ^2^ test. Differences by age and sex were examined with an independent sample *t*-test (with Levene). Pearson correlation analysis (with a z-test) was performed to explore the relationship between the factors being measured. A model-based approach, latent profile analysis (LPA) (Collins & Lanza, 2010), was applied to identify distinct social problem-solving profiles. LPA models the probability distributions of observed variables within each latent class to detect homogeneous subgroups within the data. To determine the optimal number of profiles, models with one to five latent profiles were tested. The selection of the best-fitting model was based on several statistical indicators: the Akaike Information Criterion (AIC), Bayesian Information Criterion (BIC), and sample-size adjusted BIC (SABIC), where lower values indicate better model fit. Entropy, as a measure of classification accuracy, was also considered, with values closer to 1.00 reflecting clearer profile separation. In addition, the Bootstrap Likelihood Ratio Test (BLRT) was used to compare models with *k* and *k* − 1 classes; a significant *p*-value indicated that the model with more profiles provided a better fit. The final model was selected based on acceptable entropy values (>0.80), decreasing BIC, and significant BLRT results (Spurk et al., 2020). The figure created as a result of the LPA shows the estimated means of each variable across the profiles. Each profile is represented by a separate line, allowing for a direct comparison of the response patterns. The horizontal axis shows the measured variables, while the vertical axis indicates the estimated mean scores. The error bars reflect the standard errors, providing a visual cue about the precision of the estimates and the degree of overlap between profiles. An analysis of variance (ANOVA with Tukey’s post-hoc test) was used to analyze whether there is a significant difference between the means of the variables under examination in the student profiles generated.

## 3. Results

### 3.1. Age and Sex Differences

First, we analyzed the age differences between the 16- and 18-year-old groups and the differences between boys and girls within both age groups (Table 1).

The data (Table 1) show that 16-year-olds did not score significantly higher on average in any of the areas. However, 18-year-olds are more likely to display ignoring (t = –4.692, *p* < 0.000) and have a negative problem orientation (t = −3.541, *p* < 0.041). The 16-year-old boys more commonly have an impulsive problem-solving style (M_boys_ = 2.51, SD_boys_ = 0.54, M_girls_ = 2.43, SD_girls_ = 0.61, Levene = 4.426, *p* = 0.101, t = 2.068, *p* = 0.040), and girls aged 18 typically have a negative problem orientation (M_boys_ = 2.22, SD_boys_ = 0.79, M_girls_ = 2.56, SD_girls_ = 0.89, Levene = 1.370, *p* = 0.102, t = –3.220, *p* = 0.001).

### 3.2. Correlation Analysis

Correlation analysis was used to examine the relationships between the PAS and SPSI–R factors by age group (Table 2, upper part: 16-year-olds; lower part: 18-year-olds).

Among 16-year-olds (Table 2, upper part), we identified four significant links between the PAS and SPSI–R factors. Criticism is in a positive relation with impulsivity. Further, ignoring is in a positive relation with rationality and a negative relation with positive problem orientation. In addition, sabotage is also negatively related to positive problem orientation. There is a greater correlation between the variables for 18-year-olds (Table 2, lower part). Criticism is in a negative relation with rationality, while impulsivity and avoidance are in a positive relation. Ignoring is negatively related to impulsivity but positively related to both negative problem orientation and rationality. Sabotage is also positively related to both rationality and avoidance. Two significant positive relations were found for both age groups: criticism–impulsivity and ignoring–rationality. Comparing these correlations obtained for 16- and 18-year-olds using z tests, we found the strength of the relationships is not significantly different (criticism–impulsivity: z = −0.591, *p* = 0.555; ignoring–rationality: z = −0.1.30, *p* = 0.193).

### 3.3. Latent Profile Analysis

Finally, a latent profile analysis was performed to identify profiles based on SPSI–R factor scores (Table 3, Table 4 and Table 5). The prevalence of the three PAS dimensions was analyzed for these groups (Table 6).

**Figure 1:**
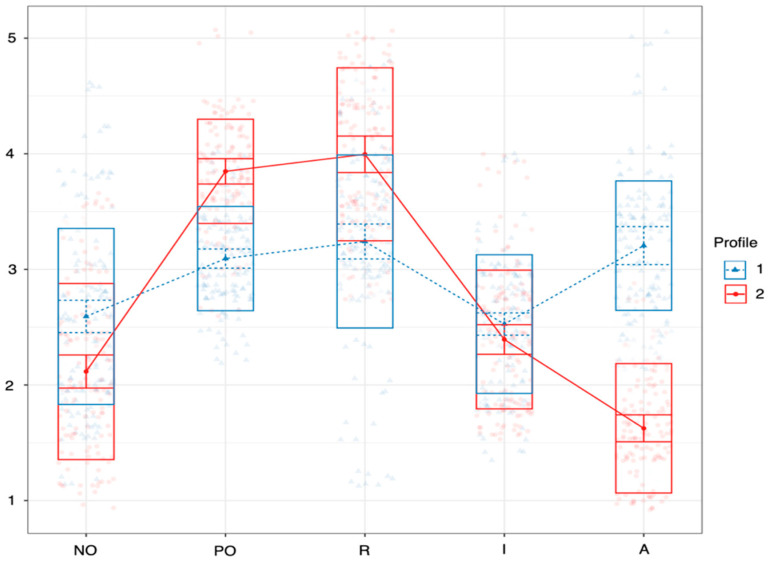
Profiles identified by latent profile analysis among 16-year-olds (NO = negative problem orientation, PO = positive problem orientation, R = rationality, I = impulsivity, A = avoidance).

**Figure 2:**
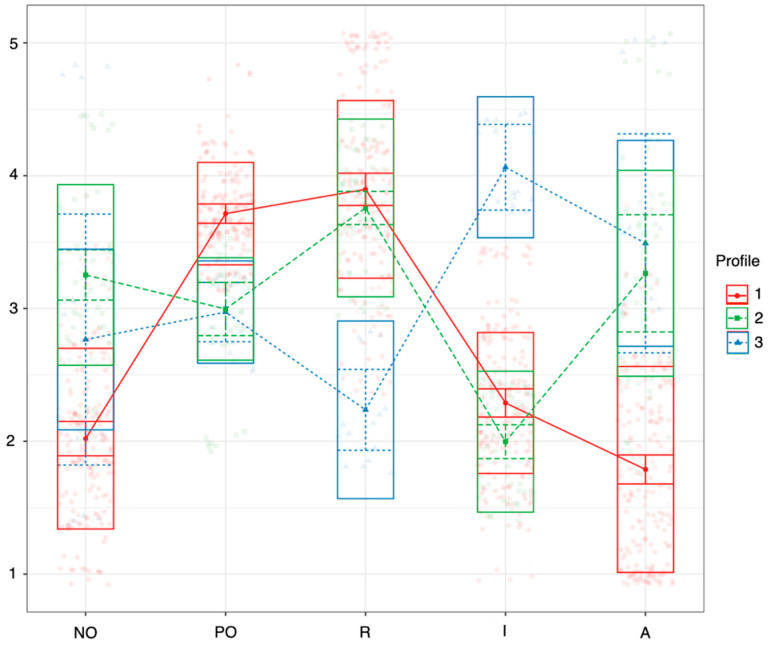
Profiles identified by latent profile analysis among 18-year-olds (NO = negative problem orientation, PO = positive problem orientation, R = rationality, I = impulsivity, A = avoidance).

For the two-profile and three-profile models, the AIC and BIC are the largest, and the closer the entropy value is to 1, the more accurate the classification of students is in each profile. Although the entropy value is highest for the three-profile model for 16-year-olds (Table 3), the number of students falling within a profile is very low, so the two-profile model was used to show the differences in the sample (Figure 1). There were three profiles distinguished for 18-year-olds (Table 4, Figure 2).

For 16-year-olds (Table 5), the two profiles are significantly different, except for impulsivity. For 18-year-olds, there is a significant separation for all factors, and only for impulsivity are the three profiles completely different. Two profiles are found in both age subsamples and are therefore labeled the same (mixed and positive rational). There is no dominant outlier in the mixed profile, with positive rationality having a high value for positive problem orientation and rational style. However, the profiles occur in different proportions in the two age groups. For 16-year-olds, the mixed profile (Profile 1, n = 136, 52.3%) and the positive rational dominant profile (Profile 2, n = 124, 47.7%) are almost equally distributed. For 18-year-olds, the positive rational dominant profile (Profile 2, n = 172, 72.9%) has the highest proportion, followed by the mixed profile (Profile 1, n = 48, 20.3%) and a much lower proportion of the impulsive avoidant dominant profile (Profile 3, n = 16, 6.8%), which is the only profile that appears in the older age group (where the impulsive and avoidant styles are both markedly high).

We tested whether there were differences between the social problem-solving profiles in terms of passive-aggressive characteristics (Table 5). Among 16-year-olds, those with a mixed profile (n = 136, 52.3%) showed slightly higher levels of passive-aggressive behaviors across all subscales compared to their positive rational peers (n = 124, 47.7%), although these differences were not tested for statistical significance within this age group. In contrast, among 18-year-olds, significant differences emerged between the social problem-solving profiles. Participants with the impulsive-avoidant profile (n = 16, 6.8%) scored significantly higher on criticism (M = 2.36, SD = 0.90) than both the mixed (M = 1.57, SD = 0.26) and positive rational (M = 1.78, SD = 0.64) groups. They also reported significantly higher levels of ignoring behavior (M = 1.75, SD = 0.48) and had elevated sabotage scores (M = 1.71, SD = 0.60) compared to the positive rational group (M = 1.58, SD = 0.62). The mixed group of 18-year-olds reported the highest level of sabotage (M = 2.01, SD = 1.13), which was significantly greater than the score of both the positive rational and impulsive-avoidant groups.

For 16-year-olds (Table 6), there is no significant difference between the two social problem-solving profiles in the application of passive-aggressive features (criticism: t = 1.15, *p* = 0.250; ignoring: t = 1.14, *p* = 0.257; sabotage: t = 0.647, *p* = 0.518). For 18-year-olds, the criticism score for the impulsive avoidant profile is significantly higher than for the other two profiles (F(2; 233) = 10. 601, *p* < 0.001). In addition, ignoring is significantly lower for the positive rational profile than for the other two profiles (F(2; 233) = 3.453, *p* = 0.042). Further, sabotage is significantly higher for the mixed profile than for the other two profiles (F(2; 233) = 4.562, *p* = 0.017).

## 4. Discussion

The communication characteristics of social problem-solving during adolescence are relatively rarely studied, and there is very little information on the emergence of passive-aggressive behaviors. This is not only because little is known about this relationship but also because this information cannot be considered when designing school-based programs for social problem-solving (e.g., Duffy, 2003). Therefore, the main aim of the present study was to explore the relationship between passive-aggressive behavior and social problem-solving among 16- and 18-year-olds.

Both Hungarian and international research (e.g., Chang et al., 2004; Kasik, 2015; Keltikangas-Järvinen, 2005) suggests that the variation in social problem-solving during adolescence shows a relatively stable pattern, with individual differences being due to personality and cultural-social influences. Between the ages of 13 and 18, negative orientation increases gradually, and positive problem orientation decreases, while avoidance is most prevalent between the ages of 14 and 16. Impulsivity remains persistently high during adolescence. In addition, rationality does not show significant changes after 15–16 years of age, and its association with some cognitive abilities (e.g., inductive and combinatorial) becomes stronger.

Based on these findings, we hypothesized that there would be no significant differences between 16- and 18-year-olds in positive problem orientation, impulsivity, and rationality but that avoidance would be more prevalent for 16-year-olds and negative problem orientation for 18-year-olds (with girls more prevalently displaying a negative problem orientation and boys more commonly showing signs of avoidance at both ages). The results only partially confirm this, which may be due to several individual background factors. We found that 16-year-olds do not score significantly higher in any of these areas, but 18-year-olds do indeed have a higher negative problem orientation. Among the 16-year-olds in the current study, boys have a more impulsive problem-solving style, and 18-year-old girls display a more negative problem orientation.

Since previous studies (e.g., Özgür et al., 2011; Schauenburg et al., 2007) have shown that passive-aggressive communication as a non-open aggressive behavior is closely related to avoidant behavior, which is most prevalent in mid-adolescence, we hypothesized that the expressions of passive-aggressive communication being investigated (criticism, ignoring, and sabotage) are more typical of 16-year-olds. The results suggest that ignoring is more common in 18-year-olds but that there are no significant differences between the two age groups for criticism and sabotage. For 18-year-olds, a much higher number of relationships were identified between problem-solving and passive-aggressive behavior, and, for the two relationships identified for both age groups (criticism–impulsivity and ignoring–rationality), there were no significant differences between the two age groups.

Based on the latent profile analysis, two problem-solving profiles were identified for 16-year-olds and three for 18-year-olds, a finding that shows similarities with a previous profile analysis in Hungarian adolescents (Fejes et al., 2023). Two of the three profiles (mixed and positive rational) were identified at both ages, while negative avoidance was only identified for 18-year-olds. Only the profiles obtained at older ages differ for passive-aggressive characteristics. The rapid formulation of negative criticism and then withdrawal from the situation is a marked characteristic of students that fall within the impulsive avoidant profile. Ignoring, avoiding, and not dealing with the other person is least characteristic of students in the positive rational profile, who have the intention of finding a solution and believing in its success. For those with a mixed profile, sabotage is the most common; that is, an apparently supportive attitude is followed by some form of obstruction of the other.

### Limitations and Future Research

Although we have obtained important information about passive-aggressive behavior among 16- and 18-year-olds and its relationship to social problem-solving, we believe that the mixed profile is a statistical product. It can be clearly seen that, although there are no outliers in SPSI–R values for this profile, sabotage implies a negative attitude towards the other, and deliberate avoidance can be assumed. Further studies will be needed to clarify this. It is also possible that the PAS used (Lim & Suh, 2022), which was developed based on adult results, is less differentially sensitive among adolescents. It is also possible that the PAS used (Lim & Suh, 2022), which was developed based on adult results, is less differentially sensitive among adolescents.

Based on these findings, we believe that future studies should focus on the development of a (non-clinical) measure of passive-aggressive behavior in adolescents. Certainly, correlation and regression relationships will provide a more accurate picture if the analysis is carried out with an instrument that allows for multiple forms of passive-aggressive behavior. This should help to identify profiles of students whose characteristics can be used to aid in the design of development school programs.

The cross-sectional study will also be worthwhile to carry out as a longitudinal study on a larger sample. A larger sample is needed to further separate profiles into distinct groups for person-centered analyses. It is also worth asking not only the students but also teachers and parents (both fathers and mothers) about the characteristics of students’ social problem-solving and communication and comparing them with the students’ self-perception.

## Figures and Tables

**Table 1:** Age differences on PAS and SPSI–R (N = 496).

PAS and SPSI–R Factor	16-Year-Olds (n = 260)	18-Year-Olds (n = 236)	Levene		*t*-Test	
	M (SD)	M (SD)	F	*p*	t	*p*
Cr	1.93 (0.75)	1.78 (0.59)	10.993	<0.000	1.117	0.052
Ig	1.41 (0.54)	1.65 (0.58)	1.046	0.307	−4.692	<0.000
Sa	1.70 (0.75)	1.76 (0.84)	2.378	0.124	−1.542	0.124
PO	3.49 (0.53)	3.47 (0.56)	0.009	0.923	0.095	0.923
NO	2.22 (0.75)	2.42 (0.86)	2.545	0.136	−3.541	0.041
R	3.64 (0.80)	3.70 (0.83)	1.999	0.158	−1.414	0.158
I	2.40 (0.59)	2.43 (0.71)	6.871	0.009	−2.622	0.109
A	2.35 (0.98)	2.31 (1.03)	0.048	0.826	0.219	0.826

*Notes*. PAS = Passive Aggression Scale; SPSI–R = Social Problem-Solving Inventory–Revised; Cr = criticism, Ig = ignoring, Sa = sabotage; PO = positive problem orientation, NO = negative problem orientation, R = rationality, I = impulsivity, A = avoidance.

**Table 2:** Correlation of variables by age (upper part: 16-year-olds, lower part: 18-year-olds; N = 496).

PAS and SPSI–R Factor	Cr	Ig	Sa	PO	NO	R	I	A
Cr	–	0.014	0.095	−0.068	0.098	0.023	0.163 *	0.022
Ig	0.002	–	0.661 **	−0.139 *	0.132	0.141 *	0.053	0.121
Sa	0.123 *	0.446 **	–	−0.198 *	0.075	0.132	0.118	0.062
PO	0.007	0.016	0.059	–	−0.199 *	0.484 **	−0.195 **	−0.631 **
NO	0.001	0.152 *	0.034	–0.439 **	–	−0.303 *	0.094	0.400 **
R	−0.301 **	0.255 **	0.122 *	0.282 **	−0.100	–	–0.315 **	−0.390 **
I	0.215 **	–0.259 **	−0.045	−0.023	0.045	−0.609 **	–	0.195 **
A	0.181 **	0.074	0.240 **	−0.477 **	0.536 **	−0.222 **	0.173 **	–

*Notes*. * *p* < 0.05; ** *p* < 0.01; PAS = Passive Aggression Scale; SPSI–R = Social Problem-Solving Inventory–Revised; Cr = criticism, Ig = ignoring, Sa = sabotage; PO = positive problem orientation, NO = negative problem orientation, R = rationality, I = impulsivity, A = avoidance.

**Table 3:** Comparison of fit statistics, indices, and likelihood ratio tests with decreasing number of groups for 16-years-olds (n = 260).

No. of Groups	Log Likelihood	AIC	BIC	cAIC	SABIC	BLRT	BLRT *p*	Entropy
2	−1358	2748	2805	2821	2754	208.56	0.01	0.834
3	−1344	2732	2810	2832	2741	27.58	0.01	0.869
4	−1323	2701	2801	2829	2712	42.92	0.01	0.821
5	−1311	2690	2811	2845	2703	23.11	0.01	0.807
6	−1251	2583	2725	2765	2598	119.52	0.01	0.858

*Notes*. AIC: Akaike Information Criterion; BIC: Bayesian Information Criterion; cAIC: consistent Akaike Information Criterion; SABIC: sample size–adjusted BIC: Bayesian Information Criterion; BLRT: parametric bootstrapped likelihood ratio test.

**Table 4:** Comparison of fit statistics, indices, and likelihood ratio tests with decreasing number of groups for 18-years-olds (n = 236).

No. of Groups	Log Likelihood	AIC	BIC	cAIC	SABIC	BLRT	BLRT *p*	Entropy
2	−1262	2556	2611	2627	2560	180.85	0.01	0.851
3	−1205	2454	2530	2552	2461	113.46	0.01	0.894
4	−1157	2371	2468	2496	2379	95.70	0.01	0.835
5	−1148	2364	2482	2516	2374	18.57	0.01	0.803
6	−1117	2313	2452	2492	2325	62.67	0.01	0.886

*Notes.* See Table 3.

**Table 5:** Results of latent profile analysis based on SPSI–R factors (N = 496).

	16-Year-Olds (n = 260)				18-Year-Olds (n = 236)					
SPSI–R Factor	Profile 1 (n = 136, 52.3%)		Profile 2 (n = 124, 47.7%)		Profile 1 (n = 48, 20.3%)		Profile 2 (n = 172, 72.9%)		Profile 3 (n = 16, 6.8%)	
	M	SD	M	SD	M	SD	M	SD	M	SD
NO	2.59 ^a^	0.83	2.12 ^b^	0.70	3.25 ^a^	0.66	2.02 ^b^	0.62	2.77 ^a^	0.33
PO	3.09 ^b^	0.41	3.85 ^a^	0.46	3.00 ^b^	0.51	3.71 ^a^	0.35	2.97 ^b^	0.27
R	3.24 ^b^	0.85	4.00 ^a^	0.64	3.76 ^a^	0.46	3.90 ^a^	0.74	2.24 ^b^	0.39
I	2.53	0.57	2.39	0.64	2.00 ^c^	0.37	2.28 ^b^	0.59	4.06 ^a^	0.31
A	3.21 ^a^	0.64	1.63 ^b^	0.41	3.26 ^a^	0.94	1.79 ^b^	0.65	3.49 ^a^	0.94

*Notes.* SPSI–R = Social Problem-Solving Inventory–Revised; NO = negative problem orientation, PO = positive problem orientation, R = rationality, I = impulsivity, A = avoidance; ^a/b/c^ = superscript letters that differ in the same row for 16-year-olds and 18-year-olds indicate statistically significant differences in means at *p* < 0.05.

**Table 6:** Characteristics of passive aggression by social problem-solving profile (N = 469).

	16-Year-Olds (n = 260)				18-Year-Olds (n = 236)					
PAS Factor	Mixed (n = 136, 52.3%)		Positive Rational (n = 124, 47.7%)		Mixed (n = 48, 20.3%)		Positive Rational (n = 172, 72.9%)		Impulsive Avoidant (n = 16, 6.8%)	
	M	SD	M	SD	M	SD	M	SD	M	SD
Cr	1.95	0.67	1.89	0.75	1.57 ^b^	0.26	1.78 ^b^	0.64	2.36 ^a^	0.90
Ig	1.50	0.53	1.42	0.55	1.82 ^a^	0.60	1.58 ^b^	0.62	1.75 ^a^	0.48
Sa	1.89	0.85	1.77	0.74	2.01 ^a^	1.13	1.52 ^b^	0.67	1.71 ^b^	0.60

*Notes*. PAS = Passive Aggression Scale; Cr = criticism, Ig = ignoring, Sa = sabotage; ^a/b^ = superscript letters that differ in the same row for 18-year-olds indicate statistically significant differences in means at *p*  < 0.05.

## Data Availability

The submitted paper includes the results of our research. The data that support the findings of this study are available from the corresponding author upon reasonable request.
